# Primaquine-thiazolidinones block malaria transmission and development of the liver exoerythrocytic forms

**DOI:** 10.1186/s12936-017-1755-6

**Published:** 2017-03-09

**Authors:** Anna Caroline C. Aguiar, Flávio Jr. B. Figueiredo, Patrícia D. Neuenfeldt, Tony H. Katsuragawa, Bruna B. Drawanz, Wilson Cunico, Photini Sinnis, Fidel Zavala, Antoniana U. Krettli

**Affiliations:** 1 Centro de Pesquisas René Rachou-Fiocruz, Av. Augusto de Lima 1715, Belo Horizonte, MG 30190-002 Brazil; 2 Faculdade de Medicina, Universidade Federal de Minas Gerais, Av. Alfredo Balena, 190, Belo Horizonte, MG 30130-100 Brazil; 3 Department of Molecular Microbiology and Immunology, Johns Hopkins Bloomberg School of Public Health, 615 N Wolfe St., Baltimore, MD 21205 USA; 4 Laboratório de Química Aplicada à Bioativos, Centro de Ciências Químicas, Farmacêuticas e de Alimentos, UFPel, Campus Universitário s/no, Pelotas, RS 98001-970 Brazil; 5 Laboratório de Epidemiologia, Fundação Osvaldo Cruz-Fiocruz Rondônia, Bairro Lagoa, Porto Velho, RO Brazil

**Keywords:** Malaria, Thiazolidinones, Sporogony, *Plasmodium berghei*, *Plasmodium gallinaceum*, Blocking malaria transmission, Exoerythrocytic stages, sporozoites

## Abstract

**Background:**

Primaquine is an anti-malarial used to prevent *Plasmodium vivax* relapses and malaria transmission. However, PQ metabolites cause haemolysis in patients deficient in the enzyme glucose-6-phosphate dehydrogenase (G6PD). Fifteen PQ-thiazolidinone derivatives, synthesized through one-post reactions from primaquine, arenealdehydes and mercaptoacetic acid, were evaluated in parallel in several biological assays, including ability to block malaria transmission to mosquitoes.

**Results:**

All primaquine derivatives (PQ-TZs) exhibited lower cell toxicity than primaquine; none caused haemolysis to normal or G6PD-deficient human erythrocytes in vitro. Sera from mice pretreated with the test compounds thus assumed to have drug metabolites, caused no in vitro haemolysis of human erythrocytes, whereas sera from mice pretreated with primaquine did cause haemolysis. The ability of the PQ-TZs to block malaria transmission was evaluated based on the oocyst production and percentage of mosquitoes infected after a blood meal in drug pre-treated animals with experimental malaria caused by either *Plasmodium gallinaceum* or *Plasmodium berghei;* four and five PQ-TZs significantly inhibited sporogony in avian and in rodent malaria, respectively. Selected PQ-TZs were tested for their inhibitory activity on *P. berghei* liver stage development, in mice and in vitro, one compound (4m) caused a 3-day delay in the malaria pre-patent period.

**Conclusions:**

The compound 4m was the most promising, blocking malaria transmissions and reducing the number of exoerythrocytic forms of *P. berghei* (EEFs) in hepatoma cells in vitro and in mice in vivo. The same compound also caused a 3-day delay in the malaria pre-patent period.

**Electronic supplementary material:**

The online version of this article (doi:10.1186/s12936-017-1755-6) contains supplementary material, which is available to authorized users.

## Background

*Plasmodium vivax* is the most widespread parasite in Asia and Americas, with three billion people estimated to be at risk of vivax malaria [1–3]. To improve the malaria control programmes and decrease malaria mortality worldwide, the World Health Organization recommends the use of primaquine (PQ), which is used to prevent *P. vivax* relapses [1]. PQ is also active against gametocytes; thus, it is used to block malaria transmission in areas approaching malaria elimination and/or facing artemisinin resistance where it is expected that transmission-blocking strategies will help to slow down the spread of resistant parasites [4]. A single 0.75 mg/kg dose of PQ is effective as a gametocytocidal agent [1]. However, PQ has restrictions because it is poorly tolerated and its metabolites cause haemolysis in patients with glucose-6-phosphate dehydrogenase (G6PD) deficiency [5]. PQ is currently the only agent approved for elimination of hypnozoites, the dormant liver stages of *P. vivax* that cause the late relapses [1].

**Fig. 1:**
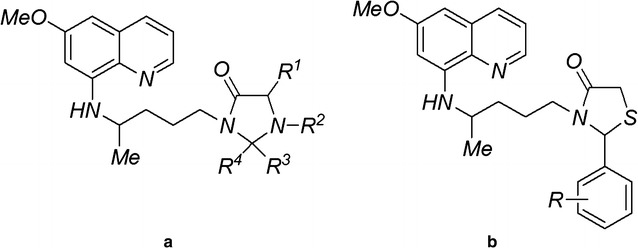
General structures of **a** PQ-imidazolidinones and **b** PQ-thiazolidinones (PQ-TZs)

The discovery that PQ-imidazolidin-4-one derivatives (Fig. 1a) exhibit transmission-blocking anti-malarial activity [6] has led to the exploration of the aliphatic terminal amino group of PQ as a precursor to the synthesis of thiazolidinones (TZ) (Fig. 1b). The thiazolidinone heterocyclic class has wide applications in the medicinal chemistry field [7] with some thiazolidinone and thiazinanone-chloroquine analogs displaying higher activity against *Plasmodium falciparum* than chloroquine (CQ) [8].

Aiming to find candidates less toxic than PQ, a total of 15 PQ thiazolidinone derivatives (PQ-TZs) were synthesized through modification of the amino-terminal group [9] and evaluated for: (i) in vitro haemolytic activity against G6PD normal or deficient erythrocytes; (ii) toxicity in vitro to hepatoma and kidney cells; (iii) blocking effect on the malaria sporogonic cycle in mosquitoes and; (iv) ability to interfere with the development of *Plasmodium berghei* liver stages in vivo and in vitro. In all assays, PQ was used as the control drug.

## Methods

### Chemical synthesis

**Fig. 2:**
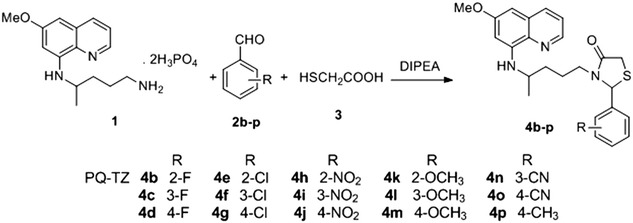
Synthetic route for PQ-TZs 4b–4p. Reaction conditions were as described [9]

The target compounds (Fig. 2) were prepared as previously described [9]. Briefly, the 2-aryl-3-[4-(6-methoxyquinolin-8-amino) pentyl]thiazolidin-4-one derivatives (PQ-TZs) 4b-p were synthesized by multicomponent one-pot reactions from PQ diphosphate 1, *N,N*-diisopropylethylamine (DIPEA), arenealdehydes 2b-p and excess of the mercaptoacetic acid 3 in refluxing toluene for 2–4 h. Primaquine (PQ), used as a control in all experiments, was primaquine bisphosphate (>98%) from Sigma). Doses were calculated using the molecular weight of the PQ-salt 455.34 g/mol.

### Human and animal ethical approvals

Human erythrocytes, normal or G6PD-deficien were collected from blood donors under the auspices of the Ethics Committee of “Centro de Pesquisa em Medicina Tropical de Rondônia–CEPEM” (CAAE-007 59912.0.0000.0011; October 9, 2012) as described in a previous publication [10]. The written consent was obtained for each new sample used in the present research.

The animal studies were approved by the Ethics Committee at the Instituto Osvaldo Cruz (FIOCRUZ, CEUA LW-23/13), and, by the Johns Hopkins University Institutional Animal Care Committee (protocol number MO13H123). Mice and chickens were anaesthesized with acepromazine 2.5 mg/kg then sacrificed by cervical dislocation. The parasitaemia was monitored in each animal starting 2 days after parasite inoculation, to determine the malaria pre-patent period (in mice subjected to sporozoite infections). The physical conditions of the animals were evaluated daily. Mice were sacrificed 3 days after the beginning of the experiment for liver stage evaluation; in other experiments, they were anaesthesized with acepromazine 2.5 mg/kg then sacrificed soon after the malaria symptoms appeared, such as anaemic blood, weight loss, ruffled fur, abnormal postural responses, reduced reflexes and reduced grip strength, coma, and convulsions. Infected chicken with an early infection were sacrificed immediately after they were used to blood-feed the mosquitoes twice in the same experiment, thus before the malaria symptoms began.

### In vitro cytotoxicity assay

The cytotoxicity of PQ and PQ-TZ compounds was assessed against cultures of: (i) a human hepatoma cell line (HepG2), received from the New University of Lisbon; (ii) a mouse hepatocyte cell line (HEPA 1-6), purchased from ATCC; and (iii) a kidney cell line from Buffalo Green Monkey Kidney (BGM) kindly provided by the Federal University of Minas Gerais. Cells were cultured in 75-cm^2^ sterile flasks in RPMI 1640 supplemented with 10% heat-inactivated fetal calf serum and 40 mg/L gentamicin, under 5% CO_2_ at 37 °C. The confluent cell monolayer was trypsinized, distributed in a flat-bottomed 96-well plate (5 × 10^3^ cells/well), then incubated for 18 h at 37 °C for cell adherence, as previously described [11]. The compounds were diluted in RPMI at a final concentration of 0.2% DMSO, and 20 µL were added at various concentrations up to 1000 µg/ML. The same diluent solution (RPMI + 1% DMSO) was used as a control in the non-treated cells. Cells were incubated for 24 h under 5% CO_2_ at 37 °C, then incubated with MTT [3-(4,5-dimethylthiazol-2-yl)-2,5-diphenyltetrazolium bromide] solution (5 mg/mL; 20 µL/well) for 3 h, in the same culture conditions. The supernatant was removed, 100 µL of DMSO was added to solubilize the formazan crystals formed, and the optical density was determined at 570 and 630 nm (background) (SpectraMax340PC^384^, Molecular Devices). Cell viability was expressed as the percentage of the control absorbance in the untreated cells after subtracting the appropriate background. The minimum lethal dose for 50% of the cells (MLD_50_) was determined as previously described [12]. A toxicity ratio between the MDL_50_ of PQ-TZ and PQ was calculated (PQ-TZ/PQ) for each drug.

### In vivo cytotoxicity assay

Selected compounds (4b, 4c, 4g, 4h, 4m, 4o, 4p) and PQ were administered to normal mice orally at a single dose. Different doses were evaluated (100, 50 and 25 mg/kg). Animal survival was observed during the following 30 days as described [13]. When the mice developed malaria, they were euthanized soon after the first symptoms appeared, as described above. The compounds were diluted in RPMI with 3% of DMSO; the control group was treated with the same vehicle.

### *Plasmodium berghei* and *Plasmodium gallinaceum* sporogonic assays

Mice were inoculated intraperitoneally (i.p.) with 10^6^ *P. berghei* (ANKA strain) infected red blood cells. Parasitaemia was monitored using Giemsa-stained blood smear and examined for the presence of gametocytes. When present, a blood sample was examined to determine the presence of exflagellating gametocytes. *Anopheles stephensi* mosquitoes were blood-feed from mice whose blood showed more than two exflagellating centres per field. The same infected mice were used twice, at time 0 h, before drug administration (control), and 2 h after receiving the drug by gavage. The drug was administered right after the first feeding. The control and experimental mosquito groups were dissected 12 days after the blood meal in mice. Midguts were removed, stained with 0.5% mercurochrome, and oocysts were counted by light microscopy. Inhibition of sporogony was calculated based on the oocyst numbers in the control mosquitoes (0 h) considered as 100% infection, and in the test mosquitoes blood-fed 2 h after drug treatment.

Chickens (1 week old) were inoculated with *P. gallinaceum*-infected erythrocytes. When parasitaemia was increasing but still below 10% they were fed-upon by *Aedes fluviatilis* mosquitoes, which were dissected at days 6–7 after the infectious blood meal. The control mosquito group was exposed to the infected chickens before drug treatment (time 0 h); the test animals were fed by mosquitoes twice, at time 0 h (control) and 4–6 h after drug treatment, as described previously [14, 15].

In all cases, PQ-TZ derivatives were tested at 50–100 mg/kg while PQ, which is more toxic, was tested at 15 mg/kg (non-toxic). PQ was diluted in water and the PQ-TZs were diluted in RPMI with 3% of DMSO, the control group was tread with the same vehicle.

The criteria used to evaluate the drug ability to block malaria sporogony was based on examination of 20 mosquitoes in each group. The percentage of infected mosquitoes (prevalence) and the mean number of oocysts per group (intensity) were calculated. A compound was considered partially active when the number of oocysts and mosquitoes infected was reduced by at least 50%, in comparison to the non-treated control, and as active when 100% reduction occurred.

### In vitro liver stage development of *Plasmodium berghei*

*Plasmodium berghei* (ANKA) sporozoites freshly isolated from salivary glands of infected *An. stephensi* mosquitoes were counted in a haemocytometer and diluted to the appropriated concentration. Ten thousand sporozoites in 0.4 mL of complete Dulbecco modified Eagle medium (DMEM) supplemented with 10% foetal bovine serum (FBS) and 40 mg/L of penicillin/streptomycin, were added to each well of a 6-well culture plate containing semi-confluent Hepa 1-6 cells seeded 1 day earlier. Drugs at 10, 1 and 0.1 µM, freshly diluted in 0.05% DMSO were added to the plates 3 h after sporozoite infection of cell cultures. The vehicle was used as a control (0.05% DMSO). The cultures were allowed to grow at 37 °C in 5% CO_2_ for 48 h. The culture medium was changed every 12 h, and fresh compounds were added at the same concentration, to maintain inhibitor pressure throughout the growth period. After 48 h, the cells were fixed with 4% paraformaldehyde, permeabilized with cold methanol overnight, blocked with 1% bovine serum albumin (BSA) in PBS for 2 h, then incubated for 1 h with monoclonal antibody 3D11 [16], directed against the circumsporozoite protein of *P. berghei*. The plates were washed three times with PBS, 0.05% Tween 20 (PBST), incubated with secondary antibody coupled to Alexa 594 (Life Technologies) for 1 h, washed three times with PBST, and mounted using prolong reagent containing DAPI (Life Technologies). Images of liver-stage parasites were captured using a fluorescence microscope (Nikon 90i). The Pro-Plus program (Media Cybernetics) was used to measure the size of the liver-stage parasites [16].

### In vivo parasite liver load assay in rodent malaria

Mice were drug-treated by oral route, on days −3, 2, 1 and 0, where day 0 corresponds to the day of sporozoite infection by intravenous route. The parasite liver load was evaluated 42 h after sporozoite inoculation, when the mice were euthanized, their livers were harvested, and total RNA extracted using Trizol reagent (Thermo Fisher Scientific, MA, USA). Reverse transcription with 4 μg of RNA was performed to obtain cDNA. In a real-time PCR mix of 50 μL, a cDNA equivalent of 0.5 μg RNA was used. The real-time PCR mix also contained primers specific for the *P. berghei* 18S rRNA and SYBR green dye. Real-time PCR was performed in a Bio-Rad I cycler, and the copy numbers were calculated using a known amount of a standard plasmid having the amplification target sequence [17]. Parasite growth inhibition was calculated by dividing the 18S rRNA copy number of the experimental group by that of the untreated control group.

### Prepatent period of malaria and survival assays

Mice were treated with 4M PQ-TZ at a dose of 50 mg/kg by oral route on days −3, −2, −1 and 0, where day 0 corresponds to the day of sporozoite inoculation. Ten thousand salivary gland sporozoites were inoculated by intravenous route and the parasitaemia and survival were followed for 30 days. A total of 5 mice were used per group. As described above, the mice were anaesthesized with acepromazine 2.5 mg/kg, then sacrificed soon after the malaria symptoms appeared.

### In vitro haemolysis assay

Experiments were performed with both human and mouse erythrocytes. Human erythrocytes (2% haematocrit) from normal or G6PD deficient donors were incubated with test and control compounds, diluted in a 0.2% (v/v) DMSO solution to 15–1000 µg/mL, at 37 °C for 2 and 24 h in a shaking water bath. To look at whether metabolites of the PQ-TZs had haemolytic properties, blood samples were taken from the mice, by retro-orbital blood collection, 15 min to 2 h after the oral treatment of the animals with PQ (15 mg/kg), with the PQ-TZ (100 mg/kg); blood from not treated animals were used as a control. Twenty microliter of serum were incubated with human erythrocytes, during 2 h at 37 °C. The mixtures were centrifuged at 1000*g* for 10 min, and the absorbance of the supernatants was measured at 540 nm in an ELISA plate reader (SpectraMax340PC384, Molecular Devices). The haemolytic rate was calculated using 0.05% saponin, as positive control for haemolysis of human erythrocytes, considered as 100% [18].

The diagnosis of G6PD deficiency was confirmed using a technique based on the principle that haemoglobin oxidized to methaemoglobin by the action of sodium nitrite is converted by the enzyme in the presence of methylene blue [19]. The final brown colour demonstrates a sample positive for G6PD deficiency, while a bright red colour is indicative of a normal sample. In the present study, the RBC samples used were of four patients with G6PD deficiency first diagnosed by one of the authors (THK), as described before [10].

## Results

### Primaquine derivatives are not as toxic as primaquine

To evaluate the toxicity of PQ derivatives on nucleated cells in vitro, HepG2 cells, a human hepatoma cell line, and a monkey kidney cell line (BGM), were incubated with the compounds for 24 h. Cell viability was measured by MTT, a colorimetric assay for assessing cell metabolic activity and the drug concentration that killed 50% of the cells (MDL_50_) was calculated. The MDL_50_ value of the PQ derivatives was compared with PQ, showing that all 14 PQ-TZ tested were less toxic than PQ; the compounds 4d, 4j, 4k, 4l, 4m, 4o and 4p were 20 times less toxic than PQ in HepG2 and over 30× less toxic to BGM cells (Table 1).

**Table 1:** Cytotoxicity of 15 primaquine derivatives (PQ-TZ **4b** to **4p**) to human hepatoma cells (HepG2) and monkey kidney cells (BGM), as compared to primaquine

Compound	R	Cytotoxicity (MDL_50_ in μM)		Toxicity ratios (MDL_50_) PQ-TZ/PQ	
		HepG2	BGM	HepG2	BGM
**4b**	2-F	NT	NT	–	–
**4c**	3-F	1302 ± 1 08	550 ± 43	4.4	2.7
**4d**	4-F	≥6831	≥6831	23.3	33.8
**4e**	2-Cl	≥2193	≥2193	9.4	10.8
**4f**	3-Cl	641 ± 31	252 ± 24	2.7	1.2
**4g**	4-Cl	3984 ± 116	NT	17	–
**4h**	2-NO_2_	1463 ± 1 05	1312 ± 145	6.3	6.5
**4i**	3-NO_2_	536 ± 17	523 ± 210	2.3	2.6
**4j**	4-NO_2_	≥6651	≥6651	28.5	32.9
**4k**	2-OCH_3_	≥6833	≥6833	29.3	33.8
**4l**	3-OCH_3_	≥6651	≥6651	28.5	32.9
**4m**	4-OCH_3_	≥6644	≥6644	28.5	32.9
**4n**	3-CN	≥2242	≥2242	9.6	11.0
**4o**	4-CN	≥6726	≥6726	28.8	33.3
**4p**	4-CH_3_	≥6651	≥6651	28.5	32.9
Primaquine		233 ± 5	202 ± 35	–	–

Drug concentration that killed 50% of cells using the MTT assay as a readout

*NT* not tested

The toxicity of the PQ derivatives was also evaluated on human erythrocytes, aiming to see if these compounds have different levels of toxicity to normal erythrocytes compared to G6PD deficient erythrocytes. Different concentrations (15–1000 µg/mL) of PQ-TZs (4b, 4c, 4d, 4e, 4f, 4g, 4h, 4i, 4j, 4k, 4l, 4m, 4n, 4o and 4p) were incubated with human erythrocytes, and the rate of haemolysis evaluated. These compounds were not haemolytic to normal erythrocytes or to G6PD deficient erythrocytes, while PQ was haemolytic, even at the lowest dose tested (Table 2). In addition, sera from drug-treated mice were tested for haemolysis, to determine if these PQ-TZs produce toxic metabolites as PQ does. Sera were collected between 15 min up to 2 h after treatment of mice with PQ (15 mg/kg) or with PQ-TZ (100 mg/kg), and incubated with the normal or G6PD deficient erythrocytes for 2 h (Table 2).

**Table 2:** Hemolysis (%) of normal and G6PD deficient erythrocytes after 2 h incubation with sera from mice dosed with primaquine (15 mg/kg) or PQTZ (100 mg/kg)

Erythrocyte samples	Hemolysis (%) by time between mouse dosage and serum collection				
	15 min	30 min	45 min	60 min	120 min
Primaquine					
Normal	0 ± 0	22 ± 12**	32 ± 10**	30 ± 16**	10 ± 11**
G6PD deficient	0 ± 0	48 ± 2**	54 ± 12**	43 ± 22**	36 ± 13**
PQTZs (**4b**, **4c**, **4d**, **4e**, **4f**, **4g**, **4h**, **4i**, **4j**, **4k**, **4l**, **4m**, **4n**, **4o** and **4p**)					
Normal	0 ± 0	0 ± 0	0 ± 0	0 ± 0	0 ± 0
G6PD deficient	0 ± 0	0 ± 0	0 ± 0	0 ± 0	0 ± 0
Not treated					
Normal	0 ± 0	0 ± 0	0 ± 0	0 ± 0	0 ± 0
G6PD deficient	0 ± 0	0 ± 0	0 ± 0	0 ± 0	0 ± 0

Mean and standard deviation of three different animals

** Statistical differences as compared to not treated controls are indicated by an asterisk (p < 0.05, by Mann–Whitney test)

All mice (total of three mice per group) survived until the end of the experiment. After the blood collection, they were anaesthesized with acepromazine 2.5 mg/kg then sacrificed by cervical dislocation. Only sera from PQ-treated mice caused haemolysis: about 48% haemolysis was observed when sera were collected 30 min following treatment. Sera collected 120 min after treatment with PQ had a lower haemolysis rate, likely from the lower concentration of circulating toxic metabolites.

The in vivo toxicity of PQ and PQ-TZs compounds was tested in mice receiving the drugs administered by gavage, 5 mice per group. PQ in a single dose of 100 or 50 mg/kg was fatal on the second day for all mice. The 25 mg/kg dose was not toxic and all mice survived for up to 30 days. Compounds 4c, 4g, 4m and 4o administered as a single dose of 1000 mg/kg caused no death within the 30 days of observation. The compounds 4b, 4m and 4p were given in a single dose of 500 mg/kg, and all mice survived for up to 30 days, the last day of observation. The animals were anaesthetized with acepromazine 2.5 mg/kg and sacrificed by cervical dislocation on day 30th (end of the experiment).

### Primaquine derivatives block *Plasmodium* transmission to mosquitoes

**Fig. 3:**
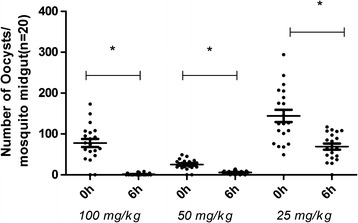
*Plasmodium gallinaceum* oocyst numbers in midguts of *Aedes fluviatilis.* Mosquitoes fed on the same infected chicken at time 0 h (before treatment) or 6 h after treatment with compound 4m, which was administered orally. For each indicated time point, 20 mosquitoes were dissected. All doses significantly reduced oocyst numbers (p < 0.001), calculated by Mann–Whitney test

Primaquine PQ-TZ derivatives were tested for their ability to inhibit oocyst formation in both rodent and bird malaria models. *An. stephensi* mosquitoes were allowed to feed on *P. berghei*-infected mice immediately before drug treatment of mice (control groups) or 2 h after treatment (test group). *Aedes* mosquitoes were fed on *P. gallinaceum* infected chickens (control groups) immediately before or after drug-treatment (test groups). Mosquito midguts were evaluated for two parameters: (a) the presence of oocysts and (b) the number of oocysts per mosquito, as compared to the control mosquito groups fed on the same animal immediately before drug treatment (considered as 100%). Thus, the effect of PQ and the test compounds on both prevalence and intensity of infection could be quantified and compared. The infected mice or chickens were fed-upon by *Anopheles* or *Aedes* mosquitoes, respectively, before drug treatment (time = 0 h) or after drug treatment (2 h in mice or 6 h in chickens). All animals (mice or chickens) after the mosquitoes fed on them were anaesthesized with acepromazine 2.5 mg/kg then sacrificed by cervical dislocation. The mosquito midgut infection was assessed 12 days later for *P. berghei* or 6–7 days later for *P. gallinaceum*. By comparing oocyst numbers in mosquitoes blood-fed on the same animal before and after drug administration, each animal served as its own control. In the *P. berghei* model, among the nine PQ-TZs tested, five (4b, 4c, 4g, 4m and 4o) completely inhibited oocyst formation. Four of these five active compounds were also active in the *P. gallinaceum* model (4c, 4g, 4m and 4o). In contrast to its strong activity in the rodent model, compound 4b was not active in *P. gallinaceum* (Table 3). Compound 4m, the only one tested in lower doses (50 and 25 mg/kg) significantly inhibited the sporogonic cycle (p < 0.001) of *P. galllinaceum* (Fig. 3).

**Table 3:** Oocyst number of *P. gallinaceum* in *Aedes* and *P. berghei* in *Anopheles*. Mosquitoes were allowed to blood feed on the infected vertebrate hosts before and after treatment with primaquine derivatives (**4b** to **4p**) or primaquine (15 mg/kg)

Compound tested (50 mg/kg)	% *Aedes* infected (% reduction)	% *Anopheles* infected (% reduction)
**4b**	80 (11%)	0 (100%)
**4c**	10 (89%)	0 (100%)
**4d**	80 (6%)	86 (4%)
**4e**	89 (0%)	NT
**4f**	95 (5%)	NT
**4g**	45 (50%)	0 (100%)
**4h**	90 (0%)	86 (0%)
**4i**	70 (13%)	NT
**4j**	75 (0%)	NT
**4k**	95 (5%)	NT
**4l**	84 (16%)	NT
**4m**	45 (55%)	0 (100%)
**4n**	95 (0%)	71 (30%)
**4o**	15 (85%)	0 (100%)
**4p**	75 (6%)	86 (0%)
Primaquine	0 (100%)	0 (100%)

The % reduction of sporogony was calculated in relation to the control mosquitoes blood fed on the same animal host immediately before treatment

### Inhibition of the exoerythrocytic stage development in vitro and in vivo

Anti-malarial drugs are designed to target the symptomatic blood stages, usually not exhibiting activity against exoerythrocytic parasites. PQ does eliminate the liver stage parasites [1] and is the only licensed drug that eliminates hypnozoites, the dormant liver stages of *P. vivax*. Selected PQ-TZs were tested for their ability to inhibit the *P. berghei* early exoerythrocytic form (EEF) development in vivo, the only EEF available for tests at our laboratories.

**Fig. 4:**
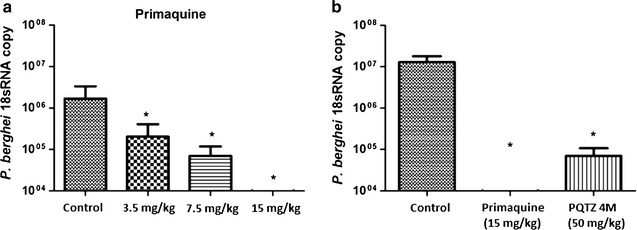
*Plasmodium berghei* liver parasite burden 42 h after intravenous inoculation with sporozoites. Parasite numbers at 42 h post inoculation were measured by quantitative PCR in mice treated with PQ (**a**) or PQ-TZ 4m (**b**). *p < 0.05 by Mann–Whitney test

Five mice were used per group. They were treated by oral route for three consecutive days (50 mg/kg per day) with PQ-TZ 4f, 4h, 4i, 4j, 4n and 4m, then they were inoculated by intravenous route with freshly isolated sporozoites and 42 h later their parasite liver load (EEF) was evaluated. The animals were anaesthetized with acepromazine 2.5 mg/kg then sacrificed by cervical dislocation, their livers were harvested and the parasites measured by real time PCR. As shown in Fig. 4, compound 4m reduced the liver parasite burden by 95% in comparison with untreated controls. Nonetheless, PQ was more active, inhibiting liver parasite burden by 75, 93 and 99%, at lower doses (3.2, 7.5 and 15 mg/kg respectively). The other compounds were inactive (Additional file 1).

**Fig. 5:**
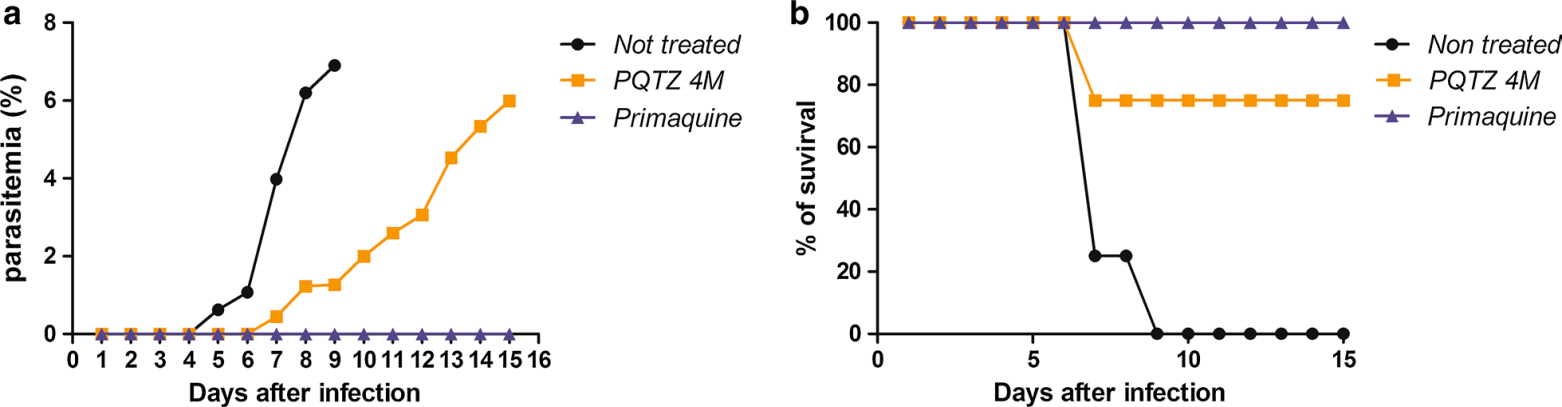
Effect of PQ-TZ on parasite burden and survival of infected mice. Mice (5 per group) were treated with PQ-TZ **4m** (50 mg/kg) or primaquine (15 mg/kg), or not treated prior to inoculation of 10,000 *P. berghei* sporozoites. Mice parasitaemia was followed by blood smears (**a**) as well as survival (**b**) for 15 days (**a**)

To determine whether the decrease in liver EEF seen in PQ-TZ 4m treated mice was due to fewer parasites becoming mature exoerythrocytic stages or to all parasites being unable to complete liver stage development, mice were inoculated with *P. berghei* sporozoites and treated with PQ or PQ-TZ 4m. All mice were followed for development of the blood stage infection. Blood smears prepared on day 3 post sporozoite infection, until day 15, had their parasitaemia determined in Giemsa-stained thin smears. Complete protection is defined as the absence of parasites in blood smears for 2 weeks post-challenge with sporozoites. The onset of blood stage parasitaemia in mice treated with PQ-TZ 4m was delayed by 3 days, compared to untreated controls. This result indicates that PQ-TZ 4m reduced but did not completely inhibit the development of infectious liver stage parasites and that some hepatic EEF were still able to initiate a blood stage infection. Nonetheless, this 3-day delay suggests that the sporozoite load was reduced by at least 100-fold. Consequently, PQ-TZ 4m treated mice also showed increased survival compared to controls (Fig. 5a, b). All five animals treated with PQ survived until the end of the experiment, when they were anesthetized with acepromazine 2.5 mg/kg and then sacrificed by cervical dislocation. Six mice were euthanized before the end of the experiment soon after malaria symptoms appeared: four non-treated control mice were euthanized on day 7 and 1 on day 9 post-infection, and one treated with PQ-TZ 4m was euthanized on day 8 due to appearance of malaria symptoms. Mice were anaesthetized with acepromazine 2.5 mg/kg and then sacrificed by cervical dislocation. The other mice survived until the end of the experiment and did not present malaria symptoms.

**Fig. 6:**
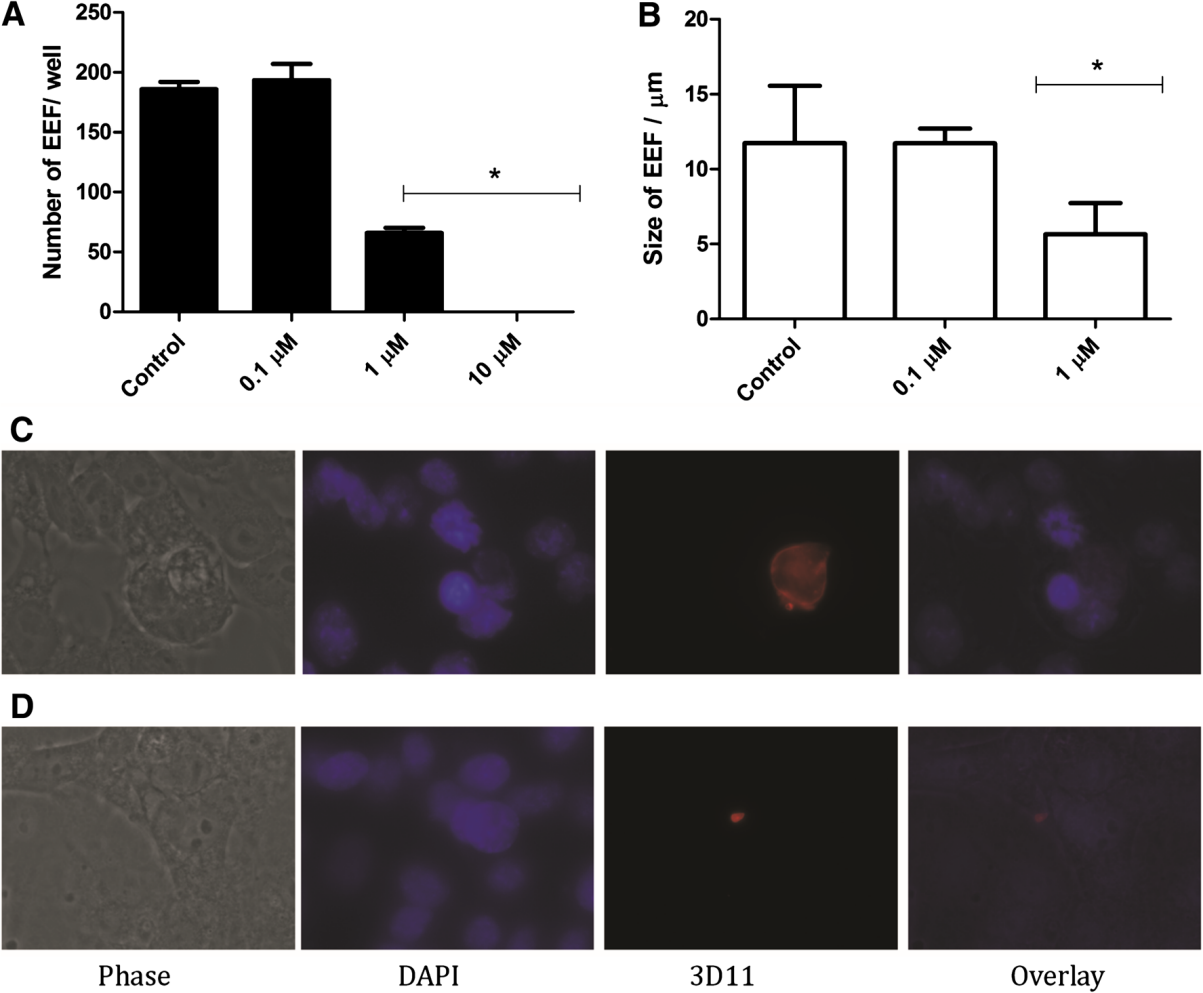
Effect of PQ-TZ 4m on infected hepatocytes in vitro. Number (**a**) and size (**b**) of the exoerythrocytic forms of *P. berghei* (EEF) in cultures of mouse hepatocytes (cell line Hepa1-6), in the presence of compound PQ-TZ **4m** at 0.1, 1 and 10 µM concentrations. Representative EEFs in control (**c**) or 10 µM PQ-TZ 4m-treated Hepa1-6 cells (**d**). The same image is shown for each in phase, stained with DAPI, and, stained with mAb 3D11 against the developing EEF. **p < 0.05 by Mann–Whitney test. Need *size bars*

We then used hepatocyte cultures to determine whether PQ-TZ 4m gave rise to fewer or smaller EEFs in vitro. Hepatoma cultures infected with 10,000 *P. berghei* sporozoites (ANKA strain) were incubated with different concentrations of 4m for 2 days, at which point cultures were fixed and EEFs stained, counted and measured. The compound PQ-TZ 4m at 1 µM significantly inhibited both the number of *P. berghei* EEFs and their size, in agreement with the in vivo results (Fig. 6).

## Discussion

PQ is used to prevent *P. vivax* relapses and to block malaria transmission. However, the toxicity of the PQ metabolites, especially the haemolytic activity to G6PD-deficient erythrocytes [20], limits its use in humans. Furthermore, primaquine causes gastrointestinal disorders such as nausea, dizziness, and vomiting in both normal and G6PD deficient patients. Hypersensitivity reactions can also occur but are rare [20]. Tafenoquine is a candidate to replace PQ that has been evaluated in clinical trials. Tafenoquine was active in a single dose but it was also haemolytic for erythrocytes in G6PD-deficient patients [21, 22].

The role of G6PD in red cells is to provide reductive potential in the form of NADPH [23]. In G6PD-deficient red cells, the NADPH supply is adequate in the steady state but falls short upon exposure to PQ. It is possible that the PQ metabolite causes an oxidative challenge that the G6PD normal red cells would tolerate, but not the G6PD-deficient red cells. However, the stepwise sequence of the events from oxidative attack to haemolysis has not been fully elucidated [24].

Aiming to decrease malaria transmission, patients with malaria, regardless of the parasite species diagnosed, receive a single dose of PQ (0.75 mg/Kg) orally to prevent malaria transmission to mosquitoes [4, 25]. The primary motivation for the present work was to study the toxicity and anti-parasite activity of PQ-TZ derivatives using rodent and avian malaria species. Pharmacokinetic studies in humans showed that carboxy-PQ is the main PQ metabolite, a compound less active than PQ [26]. The modification on the terminal amino group of PQ improved the PQ-TZs bioavailability by blocking this metabolism. Pro-drugs of PQ [27, 28], double-drugs [29, 30] and bis-PQ [31] have been studied to prevent this metabolism, as well as to reduce its toxicity in patients who are G6PD deficient. Some PQ analogues were also synthesized as PQ-trioxaquines [30] and the chemical and enzymatic stable PQ-imidazolidinones [32, 33] (Fig. 1b), a novel type of 8-aminoquinoline anti-malarial agents.

Diverse biological activities have been described for heterocyclic thiazolidinones, including antimicrobial [34, 35], anti-fungal [36], anti-inflammatory [37], anti-tumour [38], anti-HIV [39], anti-Alzheimer’s disease as muscarinic receptor 1 agonist [40], and anti-malarial [41]. As for PQ-imidazolidinone derivatives, PQ-TZs are unlikely to act as pro-drugs because the thiazolidin-4-one ring is stable at low pH. A simple test consisting of addition of concentrated chloridric acid (1 mL) to PQ-TZs (50 mg) at 40 °C for 24 and 48 h showed the stability of PQ-TZs. Moreover, by blocking the amino free group, PQ-TZs compounds may prevent the formation of cyclic pyrrolidine [42]. Indeed, as opposed to PQ, several PQ-TZs were tested in vitro on G6PD deficient and normal human erythrocytes and caused no haemolysis (Table 2).

Several reports have described the protection of the quinoline ring at the 5 position, to avoid the formation of the toxic 5-hydroxy-PQ metabolite [27, 43–45]. Although the structure of PQ-TZs does not directly influence the 5-position of the quinoline ring, 5-hydroxy-PQ-TZ might be formed. However, others report that the modification of primary amino group decreases toxicity through unknown mechanisms [45]. Indeed, most of PQ-TZs tested were significantly less toxic than PQ against human hepatoma and monkey kidney cells in vitro, as well as to normal and G6PD deficient human erythrocytes.

There is no significant influence of the nature of the aryl substituent on the ability of PQ-TZs to inhibit *Plasmodium* sporogony in the mosquito host. However, the 4-position seems to be important for drug activity. Accordingly, this position was substituted in three of the four most active compounds (4g, 4m and 4o) (Table 3), with compound 4g resulting more toxic than 4m and 4o. The compound 4m, which contains a methoxy group, inhibited *Plasmodium* sporogony at a lower dose of 25 mg/kg; the other compounds were not tested at lower doses.

In spite of the irregular production of oocysts observed in all malaria models [46], there were statistically significant differences among the groups of mosquitoes blood-fed in animals pre-treated with the compounds considered active (4c, 4g, 4m and 4o). Although none of the compounds tested completely inhibited oocyst formation in *P. gallinaceum*, 4b, 4c, 4g, 4m and 4o inhibited oocyst formation of *P. berghei* in *Anopheles* mosquitoes, as did PQ tested in parallel in both models. One possibility to explain these results is a different PQ-TZs metabolism in mammals versus avian hosts, making the mouse model more efficient at detecting active compounds. Nevertheless, in both models PQ caused 100% inhibition of sporogony.

All active compounds in the mice model were less inhibitory against the bird malaria parasites, or they caused a lower inhibition of the oocyst prevalence. These overall results could also be explained by the longer period of time elapsed between drug administration to the chicken and the mosquitoes blood meal of 4–6 h compared to 2 h in the mice model. New studies have shown that *Plasmodium yoelii nigeriensis* is a viable model system to study malaria transmission by the New World vectors *Anopheles aquasali* and *Anopheles albimanus* [47]. However, the differences between mammal and avian models need to be better understood, especially because of the limitation of a good *Anopheles* vector for infect mice in Brazil, therefore limiting the mosquito work to the *P. gallinaceum* model in Brazil.

One PQ-TZ derivative (4m) inhibiting sporogony, also inhibited development of *P. berghei* liver stages in vitro (1 µM dose), and in vivo; a result which makes the chemical group valuable. The ability of 4m to destroy the hypnozoites of *P. vivax*, as does PQ, has yet to be determined.

## Conclusions

The main findings described here include: (i) all the 15 PQ-TZs tested were not toxic to mammalian cells in vitro, unlike PQ, nor to mice treated with high doses of the new drugs; (ii) the PQ-TZs caused no direct haemolysis to human RBCs deficient or not in the enzyme G6PD; (iii) most PQ-TZs displayed a lower toxicity than PQ to mammalian cell lines - human hepatoma cells and kidney cells from green monkey; (iv) compounds 4b, 4c, 4g, 4m and 4o completely blocked the malaria sporogonic cycle of *P. berghei* in *An. stephensi* mosquitoes compared to the control mosquitoes blood-fed in the same animal immediately prior to drug treatment; (v) four PQ-TZs (4c, 4g, 4m and 4o) administered to chickens experimentally infected with *P. gallinaceum* also inhibited the sporogonic cycle by 50–90% in *Aedes* mosquitoes blood-fed after drug treatment; (vi) compound 4m reduced *P. gallinaceum* sporogony at lower doses of 50 and 25 mg/kg; (vi) compound 4m reduced the number of EEFs in hepatoma cells and in mice; (vii) compound 4m also caused a 3-day delay in the malaria pre-patent period. Whether these active PQ-TZs will become promising new drugs to help malaria control is an open question that requires further pharmacological studies and tests against the hypnozoite forms aiming to cure the *P. vivax* late relapses.

## Additional file

**Additional file 1: Table S1.** Inhibition of *Plasmodium gallinaceum* sporogony by PQ-TZ derivatives in *Aedes* mosquitoes blood fed 6 h after oral treatment (6 h) with one compound dose in relation to control mosquitoes fed in the same chicken before treatment (0 h). **Table S2.** Inhibition of *Plasmodium berghei* sporogony by PQ-TZ derivatives in *Anopheles* mosquitoes blood fed 2 h after oral treatment (2 h) with one dose of each compound in relation to mosquitoes blood fed in the mouse before drug treatment (0 h). **Figure S1.** *Plasmodium berghei* liver parasite burden 42 h after intravenous inoculation with sporozoites, measured by quantitative PCR in mice treated with PQ-TZ.

## Abbreviations

BGM: kidney cell line from Buffalo Green Monkey Kidney
EEFs: exoerythrocytic forms
G6PD: glucose-6-phosphate dehydrogenase
HEPA 1-6: mouse hepatocyte cell line
HepG2: human hepatoma cell line
MDL_50_: drug concentration that killed 50% of the cells
PQ: primaquine
PQ-TZs: primaquine derivatives
Tz: thiazolidinones
