# Supplementary material for: Primaquine-thiazolidinones block malaria transmission and development of the liver exoerythrocytic forms
Source: Malar J. 2017 Mar 9;16:110. doi: 10.1186/s12936-017-1755-6 (PMC5345155; doi:10.1186/s12936-017-1755-6)

**Additional file 1–** Inhibition of *Plasmodium gallinaceum* sporogony by PQ-TZ derivatives in *Aedes* mosquitoes blood fed 6h after oral treatment (6h) with one compound dose in relation to control mosquitoes fed in the same chicken before treatment (0h).

| **Chicken treated with (50mg/kg)** | **% *Aedes* infected**^a^  **(% reduction)** | | **Average oocyst number**  **± SD (% reduction)** | | **Inhibition of sporogony** |
| --- | --- | --- | --- | --- | --- |
|  | **0h** | **6h** | **0h** | **6h** |  |
| **4b** | 90 | 80 (11%) | 16 ± 10 | 3 ± 3 (81%) | Partial |
| **4c** | 90 | 10 (89%) | 18 ± 8 | 2 ± 1 (89%) | Partial |
| **4d** | 85 | 80 (6%) | 21 ± 4 | 26 ± 14 (0%) | No |
| **4e** | 89 | 89 (0%) | 42 ± 29 | 38 ± 25 (10%) | No |
| **4f** | 100 | 95 (5%) | 75 ± 16 | 60 ± 19 (20%) | No |
| **4g** | 90 | 45 (50%) | 35 ± 15 | 2 ± 1 (81%) | Partial |
| **4h** | 80 | 90 (0%) | 44 ± 44 | 30 ± 22 (32%) | No |
| **4i** | 80 | 70 (13%) | 33 ± 26 | 34 ± 26 (0%) | No |
| **4j** | 60 | 75 (0%) | 42 ± 38 | 38 ± 30 (10%) | No |
| **4k** | 100 | 95 (5%) | 92 ± 13 | 91 ± 20 (2%) | No |
| **4l** | 94 | 84 (16%) | 29 ± 14 | 23 ± 22 (21%) | No |
| **4m** | 100 | 45 (55%) | 47 ± 15 | 5 ± 2 (90%) | Partial |
| **4n** | 95 | 95 (0%) | 111 ± 72 | 83 ± 19 (25%) | No |
| **4o** | 100 | 15 (85%) | 49 ± 5 | 4 ± 5 (96%) | Partial |
| **4p** | 80 | 75 (6%) | 16 ± 11 | 13 ± 9 (25%) | No |

^a^ The number of oocysts and percent of mosquitos with oocysts blood-fed at time 0 h was considered to be 100% infected. ^b^Dose of PQ chosen based on the fact that 150 mg/kg kills all the mice.

The mosquitoes blood-fed in the primaquine treated control animal did not become infected, using 15mg/kg per chicken.

**Additional file 2–** Inhibition of *P. berghei* sporogony by PQ-TZ derivatives in *Anopheles* mosquitoes blood fed 2h after oral treatment (2h) with one dose of each compound in relation to mosquitoes blood fed in the mouse before drug treatment (0h).

| **Mice treated with**  **(50mg/kg)** | **% *Anopheles* infected**^a^ | | **Average oocyst number**  **± SD (% reduction)** | | **Inhibition of sporogony** |
| --- | --- | --- | --- | --- | --- |
|  | **0h** | **2h** | **0h** | **2h** |  |
| **4b** | 70 | 0 (100%) | 22 ± 15 | 0 ± 0 (100%) | Yes |
| **4c** | 86 | 0 (100%) | 64 ± 114 | 0 ± 0 (100%) | Yes |
| **4d** | 90 | 86 (4%) | 38 ± 12 | 42 ± 31 (0%) | No |
| **4g** | 80 | 0 (100%) | 75 ± 109 | 0 ± 0 (100%) | Yes |
| **4h** | 86 | 86 (0%) | 108 ± 87 | 146 ± 89 (0%) | No |
| **4m** | 86 | 0 (100%) | 49 ± 42 | 0± 0 (100%) | Yes |
| **4n** | 80 | 71 (30%) | 49 ± 84 | 27 ± 37 (45%) | No |
| **4o** | 86 | 0 (100%) | 36 ± 39 | 0 ± 0 (100%) | Yes |
| **4p** | 80 | 86 (0%) | 48 ± 23 | 35 ± 18 (27%) | No |
| **Primaquine (15mg/kg)**^b^ | 90 | 0 (100%) | 128 ± 98 | 0 ± 0 (100%) | Yes |

^a^ The percent of mosquitos with oocysts was calculated in relation to mosquitoes blood-fed in the same mouse, immediately before drug treatment (time 0 h), considered to be 100% infected. ^b^Dose chosen based on the fact that primaquine is toxic, 150 mg/kg kills 80% of mice in two days. Compounds 4e, 4f, 4i, 4j, 4k, and 4l were not tested.

The mosquitoes blood-fed in the primaquine treated control animal did not become infected, using 15mg/kg per chicken.

**Additional file 3 Figure S1:** *P berghei* liver parasite burden 42 hours after intravenous inoculation with sporozoites, measured by quantitative PCR in mice treated with PQ-TZ.


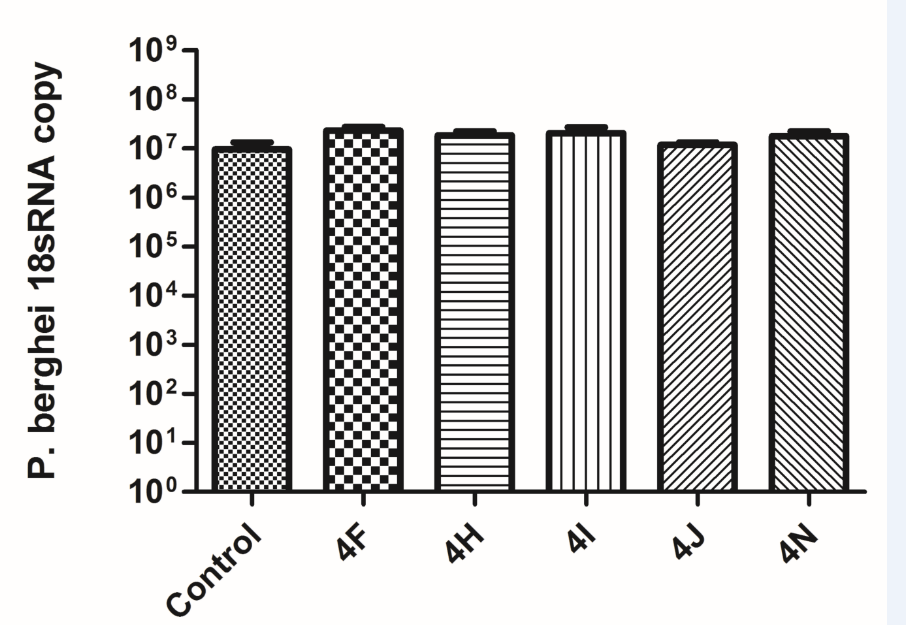

Supplement: Supplementary file 1 — Additional file 1: Table S1. Inhibition of Plasmodium gallinaceum sporogony by PQ-TZ derivatives in Aedes mosquitoes blood fed 6 h after oral treatment (6 h) with one compound dose in relation to control mosquitoes fed in the same chicken before treatment (0 h). Table S2. Inhibition of Plasmodium berghei sporogony by PQ-TZ derivatives in Anopheles mosquitoes blood fed 2 h after oral treatment (2 h) with one dose of each compound in relation to mosquitoes blood fed in the mouse before drug treatment (0 h). Figure S1. Plasmodium berghei liver parasite burden 42 h after intravenous inoculation with sporozoites, measured by quantitative PCR in mice treated with PQ-TZ. [file 12936_2017_1755_MOESM1_ESM.doc]
